# Use of inline near‐infrared spectroscopy to predict the viscosity of shampoo using multivariate analysis

**DOI:** 10.1111/ics.12536

**Published:** 2019-06-28

**Authors:** K. Haroon, A. Arafeh, P. Martin, T. Rodgers, Ć. Mendoza, M. Baker

**Affiliations:** ^1^ School of Chemical Engineering & Analytical Science University of Manchester James Chadwick Building, 2018 Booth St E Manchester M13 9SS U.K; ^2^ Unilver Research Port Sunlight Quarry Rd East Bebington C63 3JW U.K

**Keywords:** inline measurements, NIR spectroscopy, surfactant systems (personal care)

## Abstract

**Objective:**

In the personal care industry, viscosity is a critical quality attribute that influences product quality and process economics. Like many industrial liquids, personal care liquids are complex non‐Newtonian liquids made up of aqueous surfactant systems whose viscosity depends on the build‐up of micellar networks. Measuring the viscosity of complex liquids offline is easily done using benchtop rheometers and viscometers. The challenge lies in measuring the viscosity of personal care liquids online during manufacturing. Being able to track the viscosity of such products through their manufacturing cycle will not only allow for better process control but also more enhanced quality control. Therefore, the aim of this work was to investigate how proxy measurements using inline near‐infrared (NIR) spectroscopy in transmission mode can be used to predict the viscosity of shampoo. NIR spectroscopy has not, to the best our knowledge, been used to predict the viscosity of complex surfactant systems like shampoo and could significantly affect the way quality is monitored in a manufacturing environment.

**Method:**

This work focuses on viscosity changes because of differences in chloride content as salt is often used to adjust viscosity. The relationship between salt content and the viscosity of shampoo is well known following the salt curve. From an industrial perspective the region of interest for the formulation studied in this work only covers a small section of this curve. Therefore, two predictive models were developed: one covering the full range of the salt curve and another focusing on the industrially applicable region.

**Result:**

Models were produced using partial least squares (PLS) where both datasets showed some predictive ability with the concentrated region of interest showing enhanced performance [root mean square error of prediction (RMSEP) – 2.32 Pa s] compared with the larger range (RMSEP – 4.44 Pa s).

**Conclusion:**

This work provides a good starting point for developing robust predictive models for in situ viscosity measurements for shampoo manufacturing, where further work into different sources of variation and the extent of the modelling capability with regards to different formulations should be studied.

## Graphical abstract

This work presents a feasibility study into the use of inline near‐infrared spectroscopy to measure the viscosity of shampoo using partial least squares predictive modelling. As salt is commonly used to adjust the viscosity of personal care products, two models were investigated – one covering the whole salt curve and the other being more industrially relevant covering the range expected for shampoo formulations. Both models showed good predictive abilities (RMSEP: 4.44 & 2.32 Pa s respectively) with the concentrated model showing superior performance.

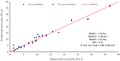

## Résumé

**Objectif:**

Dans le secteur des soins personnels, la viscosité est une caractéristique de qualité cruciale qui influe sur la qualité du produit et l’économie en matière de processus. Comme de nombreux liquides industriels, les liquides destinés aux soins personnels sont des liquides non newtoniens complexes composés de systèmes tensioactifs aqueux dont la viscosité dépend de l'accumulation de réseaux micellaires. Mesurer la viscosité de liquides complexes hors ligne est facilement effectué à l'aide de rhéomètres et de viscosimètres de paillasse. Le défi consiste à mesurer la viscosité des liquides destinés aux soins personnels en ligne pendant la fabrication. Pouvoir suivre la viscosité de tels produits tout au long de leur cycle de fabrication permettra non seulement de mieux contrôler le processus mais aussi d'obtenir un meilleur contrôle qualité. Par conséquent, l'objectif de ce travail était d’étudier comment des mesures par procuration en utilisant la spectroscopie proche infrarouge (NIR) en ligne en mode de transmission peuvent être utilisées pour prédire la viscosité du shampooing. La spectroscopie proche infrarouge n'a pas, au mieux de nos connaissances, été utilisée pour prédire la viscosité de systèmes tensioactifs comme le shampooing, et elle pourrait significativement affecter la façon dont la qualité est surveillée dans un environnement de fabrication.

**Méthode:**

Ce travail se concentre sur les changements au niveau de la viscosité en raison de différences dans la teneur en chlorure puisque le sel est souvent utilisé pour ajuster la viscosité. La relation entre la teneur en sel et la viscosité du shampooing est bien connue suivant la courbe du sel. D'un point de vue industriel, le domaine d'intérêt pour la formulation étudiée dans ce travail couvre seulement une petite section de cette courbe. Par conséquent, deux modèles prédictifs ont été développés : l'un portant sur la gamme complète de la courbe du sel et l'autre se concentrant sur le domaine applicable à l'industrie.

**Résultat:**

Les modèles ont été fabriqués en utilisant la méthode des moindres carrés partiels (partial least squares, PLS) où les deux ensembles de données ont montré un certain degré de capacité prédictive, le domaine concentré d'intérêt ayant fait preuve d'une meilleure performance (Root Mean Square Error of Prediction, RMSEP – 2,32 Pa s) par rapport à la plage de plus grande envergure (RMSEP – 4,44 Pa s).

**Conclusion:**

Ce travail fournit un bon point de départ pour élaborer des modèles prédictifs robustes servant à mesurer la viscosité in situ durant la fabrication des shampooings, mais d'autres travaux portant sur différentes sources de variation et sur l'ampleur de la capacité de modélisation de diverses formules doivent être effectués.

## Introduction

Characterizing the rheological properties of materials is of great importance in many industries in terms of product quality and process economics. In the personal care industry, viscosity is a critical quality attribute that needs to be accurately and precisely controlled. The viscosity of products like shampoo and conditioner need be thick enough to handle but fluid enough to be squeezed out of a bottle. Like all rheological problems, achieving this compromise requires an understanding of the material's microstructure, the parameters that affect the viscosity and an efficient and effective means of measuring it. Measuring the viscosity of products like shampoos and conditioners offline is well established using benchtop rheometers and viscometers 1, 2, 3, 4. Measuring viscosity in situ, however, is more of a grey area. With rheology being important across many industries, online and inline technologies have been marketed for similar applications each with benefits and drawbacks to their operation or implementation 4. Some classical instruments that had been marketed for use as online viscometers work around the same principle as their offline counterparts – for example the Brookfield TT100 measures viscosity based on the resistance of the geometry to shearing, similar to a conventional offline steady state rheometer measurement. Cambridge Piston viscometers that base their viscosity measurements on the speed that an oscillating piston moves through the liquid can be found in both laboratory and manufacturing environments. These instruments have been found to have issues with robustness, cleaning and have poor data acquisition rates 4, 5.

Other methods proposed involve measuring velocity profiles to track the viscosity of a product throughout manufacture including ultrasonic velocity profiling (UVP) 6, 7, 8 and electrical resistance tomography (ERT) 5, 9. UVP requires the use of reflective particles, in the case where they are not present tracer particles need to be added. The use of different tracer particles is a popular method of monitoring velocity profiles 10, however, addition of trace particles is undesirable in strictly regulated process environments.

Electrical resistance tomography has been shown to be applicable for measuring the velocity profile of shampoo in a batch flow set up 5. ERT exploits the idea that conductivity varies with microstructure because of local shear rates – however, many shampoo additives are conductive (i.e. viscosity modifiers and anti‐static agents) which may cause complications when monitoring the conductivity of the system.

Calorimetric studies that involve inferring viscosity from heat transfer capacity measurements have also been explored for non‐Newtonian polymers 11 and fermentation broths 12 in stirred tank reactors. A major limitation to this method is that the heat transfer area needs to be small compared with the reactor volume which could be an issue with pilot and small‐scale applications. The work was also restricted due to the power output of calibration heaters.

A recent and novel approach based on vibrating wire rheological instrumentation has been presented by Malara *et al*. 13. The uniqueness in the measurement lies in the incorporation of a fibre Bragg grating sensor that has incomparable strain sensitivity allowing for accurate measurements that cannot be accomplished using normal rheometers. Temperature and mechanical variation are known to affect the readings and therefore may pose an issue in process environments.

Another method involves tracking the decay of a fluorescence dye over time 14. This again involves adding a substance into the product which is undesirable. The dye used for this work is Thioflavin T (ThT) which is described as corrosive, irritant and toxic to humans and the environment. Another potential problem is that the dye also needs to be well incorporated into the bulk of the mixture prior to tracking – in the study conducted by Ponjavic *et al*. 14 the dye was left to dissolve for at least an hour which would increase batch turnover time.

The objective of this work is to explore the use of near‐infrared (NIR) spectroscopy (4000–12 000 cm^−1^) that uses the principle of light extinction as a result of absorption and/or light scattering, as a means of measuring viscosity *in situ*. With the introduction of chemometrics, NIR analysis has become a powerful tool 15. Many industries use predictive models based on spectroscopic data that have been processed using chemometric methods, like partial least squares regression (PLSR) and principal component regression (PCR). Many studies have been conducted on the use of near‐infrared spectroscopy combined with PLSR to predict the viscosity of petroleum products 16, 17, 18, 19, 20, 21. These studies show the potential for NIR to predict the viscosity of Newtonian liquids. Previous work in the literature have been found that use NIR spectroscopy to predict the viscosity of non‐Newtonian fluids: pharmaceutical cream 22, gravy 23, chocolate 24 and latex 25, 26. They all clearly show strong relationships between composition and viscosity where spectral variance is attributed to known compositional differences which show a distinct observable difference in NIR spectra. For this work, the only differences in the shampoo samples used are water and salt in trace amounts making comparative spectral analysis difficult. To the best of our knowledge no work has been found looking specifically at predicting the viscosity of shampoo using NIR spectroscopy.

**Figure 1:**
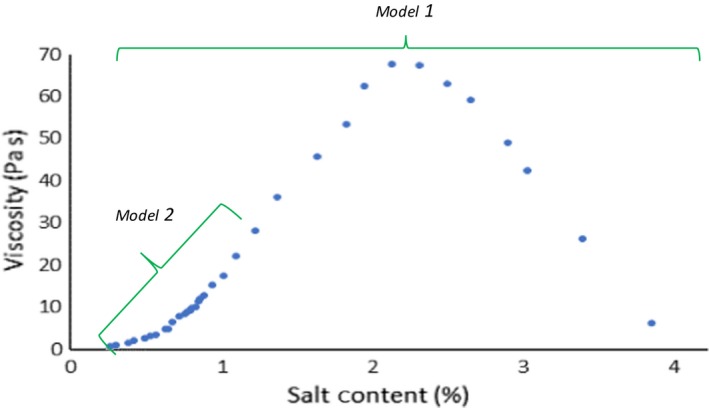
Salt curve displaying ranges for each model.

In this paper the capability of inline near‐infrared spectroscopy as a viscosity sensor for shampoo products is assessed. Shampoos are complex non‐Newtonian aqueous surfactant systems whose viscosity can be adjusted by electrolyte and surfactant concentration. This work will focus on viscosity changes because of differences in chloride content. The relationship between salt content and the viscosity of shampoo is parabolic following the well‐established salt curve 2, 27, 28, 29, 30. Increases in salt concentration induce an increase in viscosity up to a maximum value. Salt ions act to shield the head groups from repulsion between each other resulting in micellar growth and entanglement. A sharp decline with further increasing salt concentration then results because of increased branching of these entangled networks 27, 28. In the personal care industry, the region of interest for shampoo lies between 3 and 15 Pa s covering less than half of the salt curve (Fig. 1). This work investigates models produced covering the full range of the salt curve and a range that is more applicable in an industrial setting for the formulation used in this work.

## Materials and methods

### Samples

Table 1 shows the composition of the shampoo sample used. The batch was produced at Unilever (Port Sunlight, U.K.) with a formulation hole of 5.15% to adjust the viscosity of samples as necessary. The viscosity of the samples was altered by varying electrolyte (NaCl) content (and H_2_O) and ranged from 0.24% to 3.83% covering a range of 1–36 Pa s that is larger than the ideal range for shampoos (3–15 Pa s) in order to produce a robust model. Twenty‐five samples were included in the training set and four samples used for test set validation (0.62%, 0.83%, 1.08%, 2.63%) for the full salt curve model (model one). Nineteen samples ranging from 0.24% to 1.35%, were included in the training set for the industrially significant model (model two). Four samples were kept for test set validation (0.50%, 0.74%, 0.79%, 1.08%). Training and test set samples were treated in the same manner throughout the experimental and model development process. Note that only the first replicate of NIR data was used in the external validation set.

**Table 1:** Sample composition

Ingredient	Purpose	%
Sodium lauryl ether sulphate (SLES)	Primary surfactant	17.71
Cocoamidopropyl betaine (CAPB)	Secondary surfactant	5.33
Dimethiconol	Conditioning agent	1.50
Carbomer	Viscosity modifier	0.40
Sodium benzoate	Preservative	0.50
Guar	Viscosity modifier/Conditioning agent	0.25
Citric acid	pH modifier	0.10
EDTA	Chelating agent	0.05
Water	Solvent	Varies
Sodium chloride	Viscosity modifier	Varies

Although both models have small training sets (25 and 19), it is important to note that for feasibility studies, 20–30 samples are sufficient for preliminary investigations 31. Since process variances (temperature, flowrate) have not been incorporated into this model, it is expected that larger sample sets would not significantly affect the model performance at this stage. For fully developed and validated models, larger sample sets would be required while incorporating other sources of process variation.

### NIR data collection

**Figure 2:**
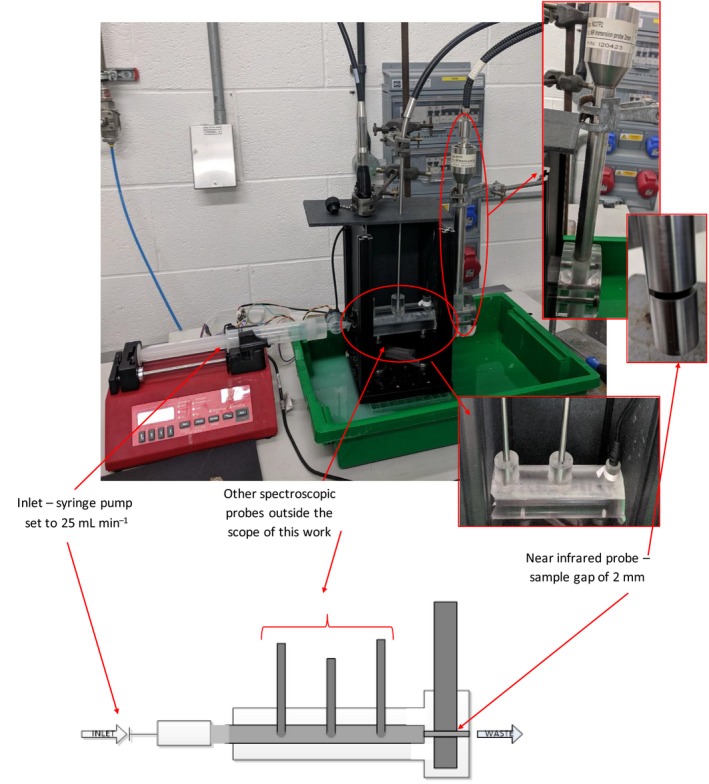
Experimental setup to simulate process conditions using a small section of perspex pipe designed to hold various inline process probes. Flow was simulated using a syringe pump able to hold 50 mL of sample and set to 25 mL min^−1^.

The spectra were acquired with a Matrix F FTNIR (Bruker, Karlsruhe, Germany) fibre‐coupled to a transmission process probe with a pathlength of 2 mm (Excalibur XP 20). The spectral range covered the whole of the NIR region (4000–12 000 cm^−1^). Spectra were acquired at 8 s^−1^ (2074 datapoints), averaged over 32 scans, with the samples at ambient temperature (≈25°C) and no temperature control being used. Samples were measured twice using a background of air. To simulate real process conditions, spectra were acquired with the sample flowing through a pipe specifically designed to ensure flow through the sample gap of the NIR probe (Fig. 2). The pipe measured 200 mm in length and samples were pumped into the system at 25 mL min^−1^.

### Multivariate analysis

Partial least squares regression was employed to extract the useful data from the spectra and produce predictive viscosity models based on reference measurements. pls toolbox chemometrics software (PLS_Toolbox_8.0.1; Eigenvector Research Inc., Wenatchee, WA, U.S.A.) that runs as a GUI in matlab (matlab 8.3, The Mathworks Inc., Natick, MA, U.S.A.) was used to perform this analysis.

Partial least squares regression decomposes both the predictor (spectroscopic) and response (viscosity measurements) data into latent variables (LV's) that best describe the respective datasets while showing the maximum covariance between them. Prior to the building of the model, spectra should be pre‐processed to minimize variances unrelated to the property of interest. For the data acquired in this study, strong baseline offsets and shifts were evident and are commonly associated with scattering effects 32. Standard normal variate (SNV) and multiple scatter correction (MSC) are techniques commonly used to remove these interferences. In mathematical terms, SNV standardizes each observation (i.e. the rows of the predictor matrix), whereas MSC uses a reference spectrum (usually the mean of the spectra) to correct the measured spectra using coefficients related to the intercept and slope. Using either of these pre‐processing techniques therefore eliminates variation because of scattering, so the subsequent spectra show only effects associated with light absorption.

Both pre‐treatments were trialled with different spectral regions of the NIR spectrum to determine the best conditions with which to continue building the model. The root mean square error statistics of calibration [root mean square error of calibration (RMSEC)], cross validation [root mean square error of cross validation (RMSECV)] and prediction [root mean square error of prediction (RMSEP)] were used as figures of merit for comparison across the various pre‐processing approaches, where RMSEC was the least significant as it gives little indication to the predictive ability of the proposed models. Cross validation was applied on the calibration samples using the venetian blinds method. In this case, samples were split systematically into five subsets, with duplicates in the same subset. This procedure prevents the ‘sample replicate trap’, where each sample is independent and leads to overfitting in the model.

Along with the RMSE statistics, residual predictive deviations (RPD) were also calculated providing a more objective metric to quantifying the model's predictive capabilities taking into account the standard deviation of the measured values and the RMSEP.

Outlier analysis involved reviewing residual statistics (*Q*), Hoteling's *T* ^2^ statistics and studying scores plots. The *Q* statistic defines the predictive ability of the model for each sample and *T* ^2^ determines how far into the model space samples lie, where potential outliers are observed outside of the 95% confidence limit. Combining this analysis with scores plot analysis allowed for real outliers to be detected and removed as necessary. After the above analysis, four samples were found to be outliers and were therefore removed.

### Reference method

The viscosity of each sample was measured using a TA AR2000 Rheometer (TA Instruments, DE, U.S.A.). Shampoos show apparent shear thinning behaviour using rotational viscometry, therefore, single point measurements were taken at a shear rate of 0.4 s^−1^ (within the Newtonian region) using a cone and plate geometry (diameter of 40 mm and angle of 4°). Samples were averaged over two measurements, temperature controlled (30°C) and covered to prevent drying during the experiment.

## Results & discussion

### Model one – full salt curve – 0.24–3.83%

#### Raw spectral analysis

**Figure 3:**
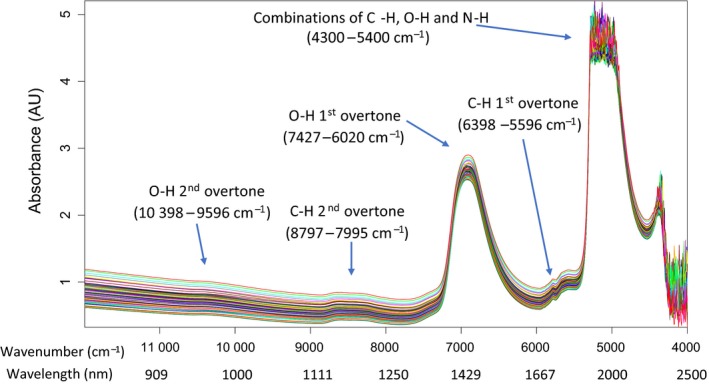
Near infrared spectra for all samples spanning the maximum spectral range possible (4000–12 000 cm^−1^).

Figure 3 displays a diagram of all the NIR spectra superimposed. As NIR is prone to overlapping peaks, the CH vibrational overtones comprise of CH, CH_2_ and CH_3_ bond absorptions, with the CH_2_ having the largest presence because of the long alkyl chains present in sodium lauryl ether sulphate. The OH overtones are representative of water molecules as water accounts for at least 65% of each sample.

The regions of interest are the first and second overtones of CH (6398–5596 cm^−1^ & 8797–7995 cm^−1^ respectively) and the second overtone of OH (10 398–9596 cm^−1^). The combination bands at the lower wavelengths (4300–5400 cm^−1^) and the first overtone of OH (7427–6020 cm^−1^) both show intensities too high to be included in calibration model. The combinations bands show very little transmission (almost total absorbance) where noise is dominating the signal and the first overtone of OH is potentially affected by nonlinearity where at this stage the interest is in correlating spectral responses to compositional changes showing linear responses.

#### Optimization of wavelength range and spectral pre‐processing

The modelling process involved determining the best pre‐treatment for the spectra to eliminate variation that is not associated with the component of interest. Clear observations of the spectra showed differences in baseline shifts commonly overcome using SNV or MSC. Both pre‐treatments were trialled individually with datasets being mean centred after to emphasize the differences in the data.

**Figure 4:**
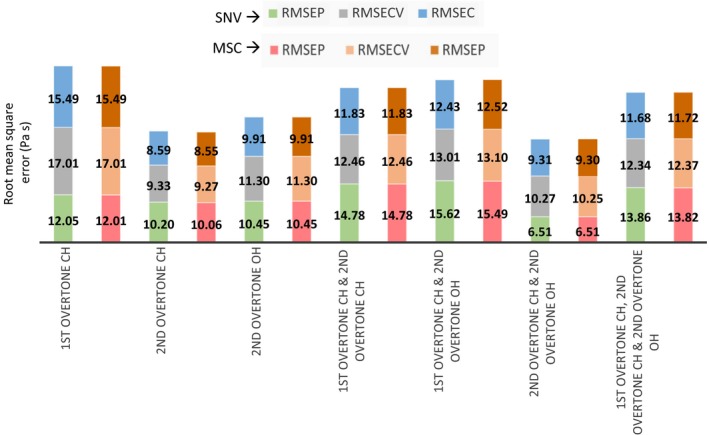
Comparison of statistical errors for model one using different pre‐processing and spectral regions.

The spectral regions trialled are detailed in Fig. 3 excluding the combination bands of OH and NH and the first overtone of OH because of reasons described earlier in raw spectral analysis. The pre‐treatments (i.e. SNV, MSC & mean centring) were applied to different combinations of the regions of interest (outlined in raw spectral analysis) where scatter correction (i.e. SNV or MSC) was followed by mean centring. The spectral regions were fixed from the start with no further variable selection or optimization. Figure 4 summarizes the models formed with different combinations of spectral regions and pre‐treatment for a single latent variable model. The variance detected in this first latent variable should represent the greatest variance in the dataset. As PLS looks for the maximum covariance between the spectral data and the offline viscosity data, the first LV should be most relevant in the prediction of viscosity. Therefore, trailing with the first latent variable provides a clear picture of where in the spectra viscosity changes are being detected and which pre‐treatments work best to emphasize the variance in these regions.

The choice of parameters was based on RMSEP, RMSECV and the RMSEC. Ideally, the errors should all be similar and low. The best prediction errors (i.e. low and equally distributed) are seen when using the second overtone of CH with SNV as the chosen pre‐processing technique.

#### Partial least squares model

**Figure 5:**
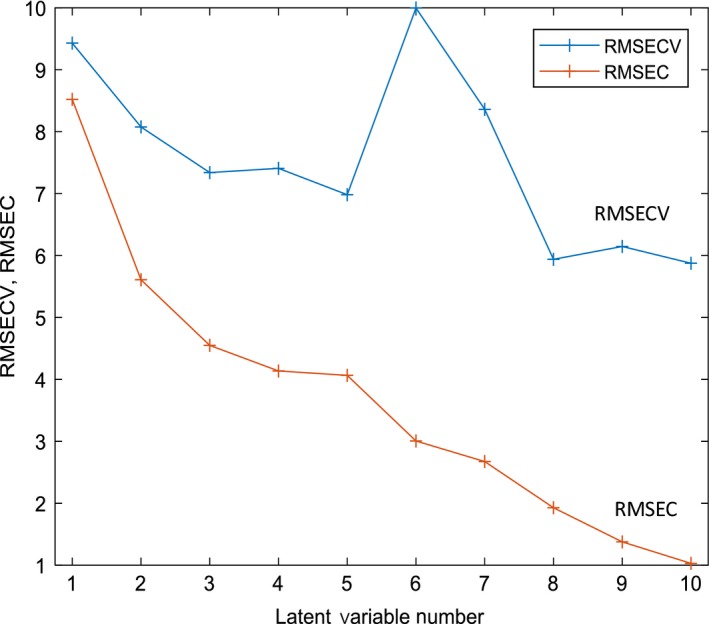
Residual error sum of squares plot for model one where the lowest RMSECV is achieved using 10 latent variables.

**Figure 6:**
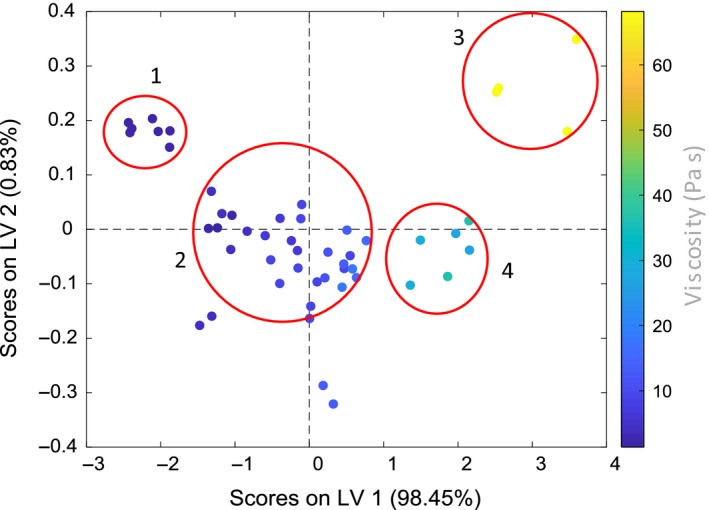
Scores plot for LV1 and LV2 showing clusters thought to be associated with the shape and structure of micelle networks.

The best model is formed using 10 latent variables (LV's) as determined by the minimum in RMSECV using Fig. 5 with RMSECV and RMSEC of 5.88 and 1.08 Pa s respectively. Using eight LV's shows comparable performance with a RMSECV of 5.94 Pa s and was chosen to prevent overfitting and noise contributions. As viscosity was explicitly included in the model, it is represented by LV1 (98.45%) as seen in Fig. 6 where the changing colour (blue to pink) represents increasing viscosity. The additional sources of variability as captured by the other LV's remain unknown, though temperature is thought to have been captured as a latent variable being known to affect NIR spectra. As temperature was not controlled or measured, the association with one of the additional LV's could not be explored further.

Figure 6 displays the scores plot for LV1 and LV2 and seems to show four distinctive clusters that may be related to the structure of the micelle networks. The clusters are numbered with increasing salt content where it is thought that in cluster one spherical micelles dominate, cluster two is associated with worm like micelles, cluster three are the highly viscous samples made up of long and highly entangled structures and cluster four entangled and branched networks with the highest salt content.

#### Validation

**Figure 7:**
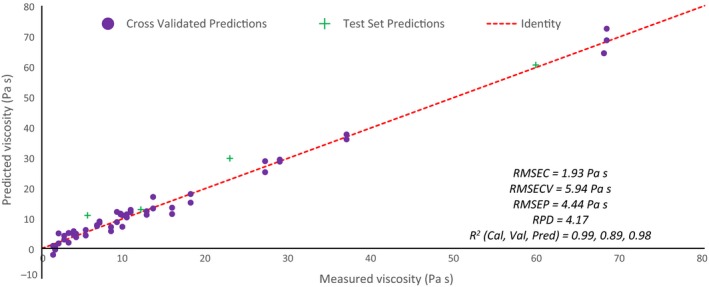
Correlation plot for model one cross validation and test set predictions.

The model was validated using cross validation (venetian blinds using five data splits leaving out 20% of the data with a sample thickness of two) and test set validation using four samples. RMSECV and RMSEP were calculated to be 5.94 and 4.44 Pa s, respectively, as seen in Fig. 7 where the dashed line represents the identity line (i.e. a perfect model). The RPD was found to be 4.17 where typically models with RPD of more than 3.0 are deemed adequate, those between 5.0 and 6.4 suitable for quality control and those above 6.4 good for process control 33. These results indicate that the model, at present, would not be suitable for quantitative analysis.

A noteworthy observation is that many of the calibration set samples are concentrated in the low viscosity range (>50%) with only a few reaching into the higher viscosities. Adding more samples of higher viscosity may improve the model. It is also worthwhile to note that there are no samples present between about 40–60 Pa s in the calibration set as they were deemed to be outliers after *T* ^2^, *Q* residual and scores plot analysis.

### Model two – industrially relevant range – 0.24–1.35%

#### Optimization of wavelength range and spectral pre‐processing

**Figure 8:**
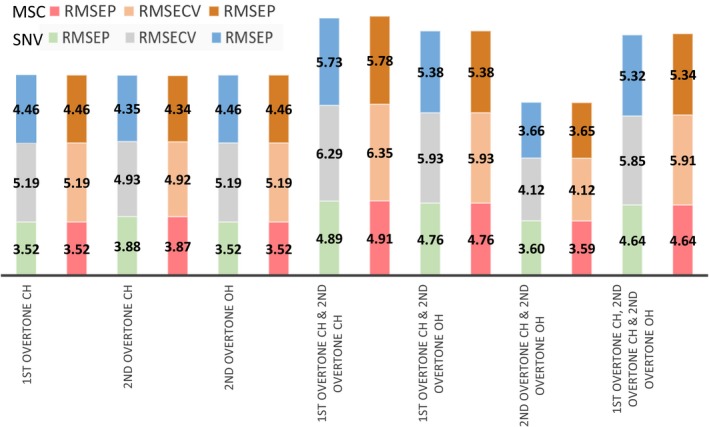
Comparison of statistical errors for model two using different pre‐processing and spectral regions.

Spectral region and pre‐processing optimization were conducted using the same method as model one. Figure 8 shows that the errors produced using SNV and MSC are comparable, therefore either pre‐treatment option could be implemented. SNV was preferred as it was also used for model one. Using the second overtone of CH and OH clearly gives the best model performance.

#### Partial least squares model

**Figure 9:**
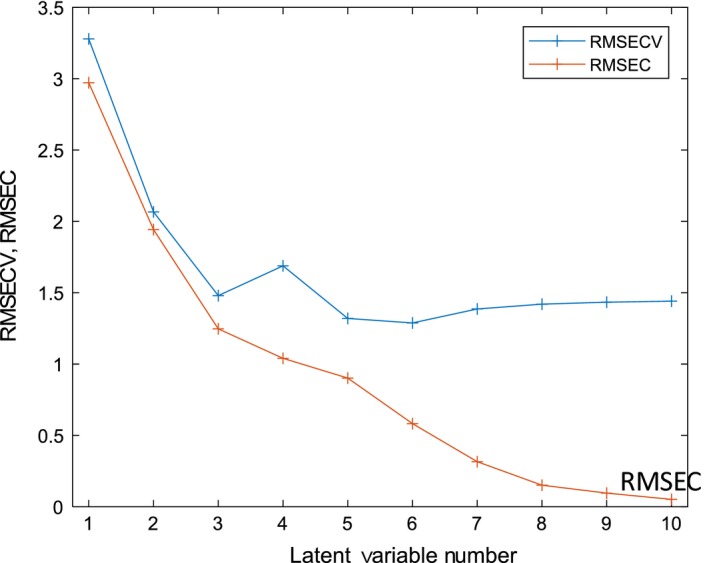
Residual error sum of squares plot for model two where the lowest RMSECV is achieved using six latent variables.

**Figure 10:**
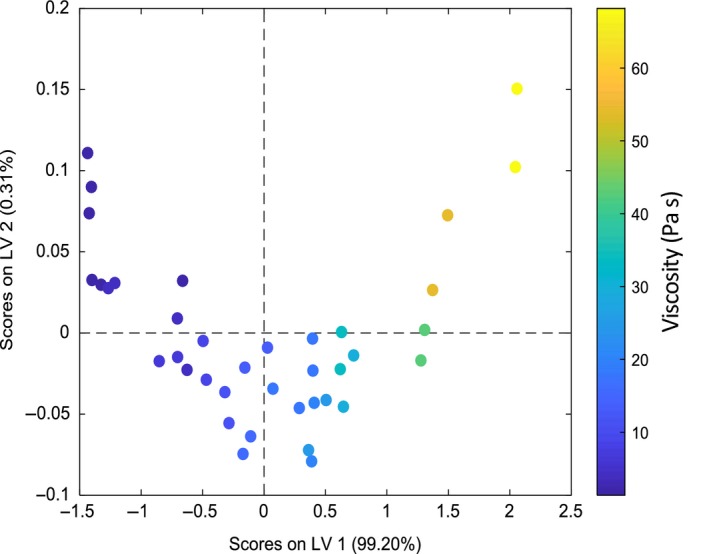
Scores for LV1 and LV2 for model 2.

The best model is formed using six LV's as determined by the minimum in RMSECV as in Fig. 9 that shows the calibration and validation errors converge using up to three variables. Increasing the number of LV's from three to six does not significantly change the error of cross validation, therefore, a three LV model was employed that captured 99.95% of the variance in the dataset. Figure 10 shows a scores plot for LV1 and LV2 where the data points are coloured according to their measured viscosity.

**Figure 11:**
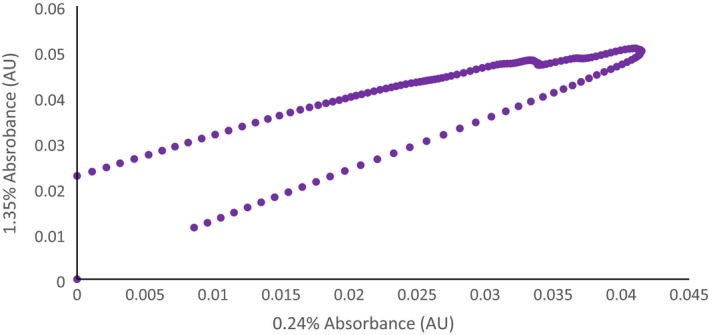
Scatterplot of absorbances for 0.24% and 1.35% salt in the second overtone of CH region – highlighting the presence of nonlinearity in this region.

The shape of scores plot (Fig. 10) shows a distinctive arch. Initially, it was thought that this is indicative of the horsehoe effect because of the distortion of the data as a result of nonlinearity in spectral regions when using absorbances covering large regions of contiguous wavenumbers 34, 35. Analysing these plots without knowledge of this effect could lead one to believe there is a connection between the high and low viscous samples because of their scoring on LV2. By taking the samples on the edge of the horseshoe (0.24% & 1.35%) and plotting their absorbances against one another for the region indicative of the second overtone of CH (Fig. 11) it is clear that nonlinearity is present in these spectral regions. Though the horseshoe effect may be affecting the shape of the plot, it may also likely be because of the dataset having a single dominant direction of variation as LV1 accounts for more than 99% of variation in the dataset.

#### Validation

**Figure 12:**
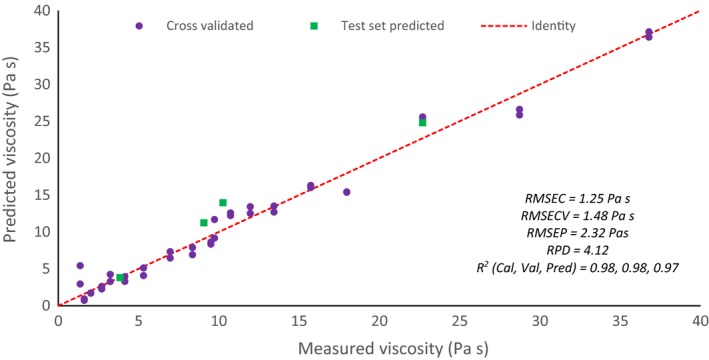
Correlation plot for model two cross validation and test set predictions.

The model was validated using cross validation (venetian blinds using five data splits leaving out 20% of the data) and test set validation using four samples. RMSECV and RMSEP were calculated to be 1.48 and 2.32, respectively, as shown in Fig. 12 where the dashed line represents a perfect model.

Model one performs best when concentrated solely on the second overtone of CH, whereas model two requires information from the second overtone of OH as well as the second overtone of CH.

The RPD for the model was calculated to be 4.12. This result shows the model is not yet good enough to be used in quality control but still provides some indication of viscosity variation. As only four samples are included in the validation set, it is thought that further development of such a model would improve its performance and therefore the RPD value.

**Figure 13:**
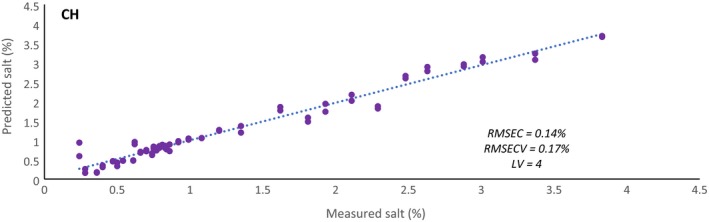
Predictive salt concentration model using the spectral region of the second overtone of CH (8797–7995 cm^−1^) where the dotted line represents the fit.

**Figure 14:**
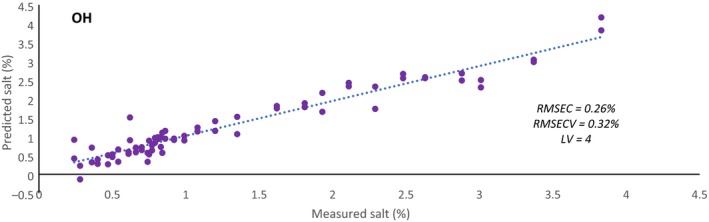
Predictive salt concentration model using the spectral region of the second overtone of OH (10 398–9596 cm^−1^) where the dotted line represents the fit.

It is thought that the information contained in the second overtone of OH may be related to salt content because of previous works found that were able to detect differences in salt in aqueous solutions using the OH overtones obtained in NIR spectroscopy 30, 36, 37. To test this idea, the data from model one were used to develop rough models based on salt content rather than viscosity using the second overtone of OH and the second overtone of CH individually. No pre‐processing techniques were investigated (note that both datasets were mean centred only) and the models developed only focused on latent variable optimization. Figures 13 and 14 show that using the second overtone of CH produces a better model with a RMSECV of 0.17% – nearly half of that compared with using the second overtone of OH (0.32%). This shows that information regarding salt content is more prevalent in the second overtone of CH but is still present in the OH region. As well as having some association with salt content, it is suspected that the variability detected in the CH spectral regions is also attributed to alignment changes of the lipophilic tails of surfactant systems. The viscosity of such systems is increased with initial addition of salt, that acts to shield the polar heads from one another allowing for tighter and increased packing of micelles. This may be influencing the alkyl chains that are becoming increasingly confined which could be affecting their vibrational motion.

The variation found in the OH overtone may be related to the quantity of bound water molecules which varies as the micelles structure evolves with increasing salt concentration. At the lower salt concentrations as micelles change from spherical to elongated and entangled more water molecules become trapped in the structure. Further increases in salt results in a fall in viscosity where water molecules become less structured as the micelles form branched networks. With moderately branched micelle networks, it is thought that the microstructure is quite chaotic and prone to unfavourable interactions between bound water and lipophilic cores that change the structure of the water 38, which could explain why only model two finds useful information in the OH spectral region as the salt concentrations used here are not high enough to induce any branching and enter into the disordered microstructural phase, however, further investigation is required to understand how the water molecules behave as a result of the evolving micelles.

## Conclusion

The development of NIR calibrations using quantitative PLS models for the inline measurement of the viscosity of shampoo has been successfully demonstrated. Two models were generated covering different viscosity ranges. Model one included samples covering the whole salt curve. Model two focused on the region that would be of significance from an industrial standpoint for the formulation used in this study. Both models showed some predictive ability with model two showing better performance. It is important to note that this study reports on preliminary work for a predictive model for the viscosity shampoo. For the model to be of use, larger samples sets would be required to ensure that the model is robust but also to ensure that validation is done properly.

Model one shows satisfactory predictive ability with a RMSECV of 5.94 Pa s and RMSEP of 4.44 Pa s. Model two shows very good errors of cross validation and prediction (1.48 & 2.32 Pa s respectively). From an industrial standpoint, these statistics show promise as a preliminary exploration into the ability of NIR spectroscopy to predict the viscosity of shampoo inline within a manufacturing environment removing the need for offline testing and therefore improving process efficiency and subsequently quality control.

Further work should involve including sources of variation encountered in manufacturing environments (i.e. batch, temperature variation). To better understand the scope of near‐infrared modelling for the viscosity of shampoo formulations, investigations into the effect of different additives and surfactant systems on NIR spectra and models should be explored. Furthermore, this study is based on viscosity changes because of salt content. Salt is one of many materials that can be used to alter the viscosity of personal care liquids. Exploring how and if NIR can be used to determine the viscosity of shampoos when modified with different materials will allow for a better understanding into the extent of variation that can be included in such predictive models. Further work will also involve exploring how (if at all) the liquid is stressed upon entering the sample gap of the NIR probe using computational fluid dynamics (CFD), which could lead to designing a new probe able to overcome this issue. This work is also being investigated using process Raman, mid‐Infrared (MIR) and nuclear magnetic resonance (NMR) spectroscopy where synergistic models are being considered.
